# Cognitive and emotional factors associated with the desire to cease non‐suicidal self‐injury

**DOI:** 10.1002/jclp.23336

**Published:** 2022-03-05

**Authors:** Nicole Gray, Penelope Hasking, Mark Boyes

**Affiliations:** ^1^ School of Population Health Faculty of Health Sciences Curtin University Perth Western Australia Australia

**Keywords:** ambivalence, behaviour, cessation, intention, nonsuicidal self‐injury

## Abstract

**Background:**

Due to cognitive and emotional differences between individuals who have and have not stopped self‐injuring, we explored these in the context of desire to stop.

**Method:**

Australian university students (*n* = 374) completed cognitive and emotional measures. Comparisons were made between those who had self‐injured in the past 12 months and those who had not, and between individuals who reported wanting to stop self‐injuring and those who did not.

**Results:**

Approximately 20% of participants did not want to stop self‐injuring. Cognitive emotional factors (psychological distress, self‐efficacy to resist, difficulties regulating emotion, interpersonal functions, and outcome expectancies) differentiated individuals who had and had not stopped, but could not explain differences in desire to stop.

**Conclusion:**

Factors associated with desire to stop are not the same as factors underlying behavioural cessation. Motivational approaches to changes in self‐injurious behaviour would be beneficial for clinicians and their clients.

Nonsuicidal self‐injury (NSSI) is the direct and deliberate damage of one's own body tissue without suicidal intent (International Society for the Study of Self‐injury, 2018). Self‐injurious behaviours include cutting, burning or scratching the skin, and self‐battery (Nock, 2010). NSSI is prevalent across clinical (20% of adult, 40%–80% of adolescent) and community (13% of adult, 17% of adolescent) populations (Kaess et al., 2012; Klonsky & Muehlenkamp, 2007; Swannell et al., 2014). Pooled estimates for NSSI among university students are placed at approximately 20%, suggesting higher rates in university cohorts compared to the general population (Swannell et al., 2014).

Individuals report engaging in NSSI for a variety of reasons (self‐punishment, avoiding suicide, communication of pain); however, the most common function of NSSI is to regulate unwanted emotional states (Klonsky & Glenn, 2009; Taylor et al., 2018). It is reported that NSSI reduces negative affect, providing relief for the individual in moments of distress, thus maintaining the behaviour through negative reinforcement (Chapman & Dixon‐Gordon, 2007; Taylor et al., 2018).

The behaviour is often, though not always, associated with psychological disorders including depression, anxiety, and posttraumatic stress disorder (Bentley et al., 2015; Kiekens et al., 2018; Moran et al., 2014; Nock & Favazza, 2009; Whitlock et al., 2013). Additionally, while not performed with suicidal intent, there is also evidence linking ongoing NSSI to future suicidal thoughts and behaviours (Asarnow et al., 2011; Hamza et al., 2012; Kiekens et al., 2018; Scott et al., 2015; Whitlock et al., 2013). There may be an increase in frequency, number of methods used, and severity of NSSI over time (Andrews et al., 2013) and, in some cases, the behaviour can require hospital treatment (Owens et al., 2016). Negative reactions to disclosure (Staniland et al., 2020), and scarring (Lewis & Mehrabkhani, 2016) may perpetuate feelings of shame over the behaviour, leading to further NSSI. The short‐term (e.g. shame, guilt; Tan et al., 2019) and long‐term (e.g. scarring; Lewis & Mehrabkhani, 2016) outcomes of NSSI may cause further emotional distress for an individual (Andrews et al., 2013; Owens et al., 2016).

Given these negative correlates, it appears reasonable to view NSSI as an undesirable behaviour, which one would want to avoid, and numerous reasons to cease NSSI are pertinent in the mind of those who want to stop (e.g., to minimise unwanted attention; unwanted scars; preserve relationships with distressed family and friends; decrease personal shame; Deliberto & Nock, 2008; Gelinas & Wright, 2013). However, many individuals view NSSI as an effective coping strategy, asserting a desire to continue the behaviour due to (a) the function it serves (affect regulation, communication, facilitating a sense of control, feelings of safety) or (b) to avoid the disappointment of re‐engaging after a period of abstinence (Hambleton et al., 2020; Kelada et al., 2017; Klonsky & Glenn, 2009; Tan et al., 2019). Additionally, in some studies, participants report experiencing fascination, pride, and pleasure over their self‐injurious behaviours (Hambleton et al., 2020). As such, the cessation of NSSI appears to be a complex process, driven by subjective perspectives on a range of factors (Shaw, 2006).

A number of studies have explored the differences between individuals who currently engage in NSSI and those who have stopped the behaviour (Andrews et al., 2013; Deliberto & Nock, 2008; Gelinas & Wright, 2013; Hambleton et al., 2020). In studies comparing individuals who have ceased self‐injuring (usually defined as no self‐injury in the past 12 months; e.g., Andrews et al., 2013; Kelada et al., 2017) with those who have not, individuals who continued to self‐injure were more likely to report using it for intrapersonal reasons (e.g. Halpin & Duffy, 2020). Not surprisingly then, continued engagement in NSSI was also associated with elevated psychological distress and difficulties regulating emotion (Whitlock et al., 2015). An expectation that NSSI will help with affect regulation is also associated with continued engagement in the behaviour (Dawkins et al., 2019). In contrast, endorsing NSSI for interpersonal reasons (e.g., peer bonding), or expecting NSSI to result in physical pain are associated with cessation of the behaviour (Halpin & Duffy, 2020). Finally, individuals who have ceased self‐injuring report higher self‐efficacy to resist the behaviour than those who continue to self‐injure (Dawkins et al., 2019; Tan et al., 2019).

Investigation into the factors differentiating individuals who have and have not ceased self‐injurious behaviour is important, however, Grunberg and Lewis (2015) highlight that changes in self‐injurious behaviour arise through changes in perceived costs and benefits of the behaviour. For example, NSSI may be beneficial as it reduces negative affect, yet may also cost the individual if the behaviour leads to negative social outcomes, shame, or regret (Hooley & Franklin, 2017). Reflected in motivational interviewing techniques, behaviour change often comes from a desire to change, following evaluations of the costs and benefits of the behaviour (Grunberg & Lewis, 2015; Kress & Hoffman, 2008; Prochaska et al., 1994).

While essential to the understanding of NSSI, an ongoing emphasis on behavior overlooks a vital precursor, the desire to act on, or cease the behaviour. Gelinas and Wright (2013) explored factors contributing to the cessation of NSSI, finding that among those who had stopped, “a desire for wellness” (p. 380) was a recurrent theme for approximately 13% of individuals (p. 380). However, no particular reasons why they had a desire for wellness (e.g., interpersonal influence, intrapersonal emotions) were noted (Gelinas & Wright, 2013). Comparably, Hambleton and colleagues (2020) reported that of the individuals who had stopped NSSI, approximately 11% reported a desire to feel healthy, and approximately 11% (not mutually exclusive) reported a desire to stop scarring, with the expectation that others would find it unacceptable. In this research there is limited understanding as to which specific factors may be driving this desire.

Many theories embedded in the substance use literature have long recognised the importance of cognition and emotion in substance use behaviours (Cox & Klinger,  1988; Koob, 2015). Previous research has explored cognitive and emotional processes related to both substance use behaviour, and desire to use substances. For example, Barkby et al. (2012) found that anxiety and depression were higher among alcohol dependent participants than nonalcohol dependent participants. Dickson et al. (2013) found that positive and negative alcohol outcome expectancies were experienced more by individuals who once drank heavily and had stopped, than those who continued to drink socially. In terms of desire to engage in substance use, Greely et al. (1993) found that positive desire for alcohol was associated with higher levels of stress and depressive affect, and lower levels of self‐efficacy to resist drinking, compared to individuals who report negative desire. The contribution of cognitive and emotional factors (e.g., emotion regulation, outcome expectancies, self‐efficacy to resist NSSI) has been repeatedly identified in the research on the cessation of NSSI (e.g., Andrews et al., 2013; Deliberto & Nock, 2008; Kiekens et al., 2017). However, research could further clarify whether the reasons for stopping NSSI are similarly driven by cognitive or emotional processes; identifying the thoughts and feelings around wanting to stop/continue may provide useful treatment and intervention targets. This study aims to explore the NSSI‐related, emotional, and cognitive factors associated with a desire to stop self‐injuring or not, and determine the extent to which these factors are (or are not) consistent with factors that differentiate individuals who have and have not ceased self‐injuring.

## 1 METHOD

### 1.1 Participants

The sample comprised 374 participants attending a total of 28 universities across Australia. The majority of these (75%) were recruited though the Curtin University School of Psychology undergraduate participation pool. All other participants were recruited via advertising through their student guild. All participants had engaged in NSSI at some point in their lives. The sample was aged between 18 and 52 (*M* = 23.58, *SD* = 4.19). Of the sample 301 (80.7%) were female, 62 (16.6%) were male, and 10 (2.7%) identified as another sex. The mean age for initial engagement in NSSI was 14 years (*SD* = 3.28). The majority of participants (*n* = 163, 45.7%) considered cutting to be their main form of the behaviour, with self‐battery (*n* = 48, 13.4%) the next most frequently reported. 318 (96.1%) participants reported experiencing pain when they self‐injured.

### 1.2 Materials

Alongside sociodemographic information (age and sex), the following measures were included in the study.

#### 1.2.1 Psychological distress

Levels of depression, anxiety, and stress were assessed using the 21‐item Depression Anxiety and Stress Scale (DASS‐21; Lovibond & Lovibond, 1995). Participants were asked how often they had experienced given symptoms in the last 4 weeks. Responses were recorded on a 4‐point Likert scale (*0* = *never; 3* = *almost always*). This measure has demonstrated convergent validity (Lovibond & Lovibond, 1995), correlating strongly with the Beck Depression Inventory (*r* = .74; Beck & Steer, 1987) and the Beck Anxiety Inventory (*r* = .81; Beck & Steer, 1990). Internal consistency has been demonstrated in previous studies (*α* = .91; Lovibond & Lovibond, 1995). Good internal consistency was also demonstrated within the present sample (depression, *α* = .93; anxiety, *α* = .87; and stress, *α* = .87.

#### 1.2.2 Difficulties in emotion regulation

Difficulties in emotion regulation were assessed using the Difficulties in Emotion Regulation Scale (DERS; Gratz & Roemer, 2004). This is a 36‐item measure assessing participants' perceived difficulties regulating emotion. Item responses are recorded on a 5‐point Likert scale (*1* = *almost never; 5* = *almost always)*. Higher scores indicate greater difficulty regulating emotion. The measure assesses overall difficulties, as well as six specific components of emotion regulation: nonacceptance of emotions; difficulties with goal‐oriented behaviours; difficulties managing impulsive behaviours; limited awareness of emotion; difficulties accessing regulation strategies; difficulties clarifying emotional experiences. The DERS has demonstrated strong construct validity alongside mood regulation scales, avoidance, and expression of emotions (Gratz & Roemer, 2004). The measure demonstrated strong internal consistency across all subscales in previous studies; Nonacceptance (*α* = .85). Goals (*α* = .89), Impulse (*α* = .88), Awareness (*α* = .80), Strategies (*α* = .88), Clarity (*α* = .84; Gratz & Roemer, 2004). Internal consistency was acceptable across the individual subscales in the current sample; Nonacceptance (*α* = .93); Goals (*α* = .87); Impulse (*α* = .89), Awareness (*α* = .84), Strategies (*α* = .91), Clarity (*α* = .87).

#### 1.2.3 Outcome expectancies

NSSI related outcome expectancies were assessed using the Non‐Suicidal Self‐Injury Expectancies Questionnaire (NEQ; Hasking & Boyes, 2018). The measure comprises 25 items asking the perceived likelihood of a given outcome when engaging in NSSI. Reponses are recorded on a 4‐point Likert scale (1 = *extremely unlikely*, 4 =* extremely likely*) across five subscales: affect regulation (e.g., “I would feel less frustrated with the world”); negative social experiences (e.g., “My friends would be disgusted”); communication (e.g., “I would get care from others”); pain (e.g., “I would feel physical pain”); and negative self‐beliefs (e.g., “I would hate myself”). Strong internal consistency across all five subscales has been demonstrated in previous studies (Dawkins et al., 2019). In the current sample, internal consistencies were adequate to excellent (affect regulation *α* = .69; negative social outcomes *α* = .86; communication *α* = .91; negative self‐beliefs *α* = .81; pain *α* = .84).

#### 1.2.4 Self‐Efficacy

Self‐efficacy to avoid self‐injury was measured using an adaptation of the 6‐item Self‐Efficacy to Avoid Suicidal Action Scale (Czyz et al., 2014). The adapted version (Hasking & Rose, 2016) measures an individual's perceived ability to resist engaging in NSSI. Six items (e.g., “How certain are you that you will not self‐injure in the future?”) are responded to on a 6‐point Likert scale (1 = *very uncertain*, 6 =* very certain*). Higher scores indicate greater perceived self‐efficacy to resist NSSI. The measure has demonstrated strong internal consistency in previous studies and can differentiate individuals with and without a history of NSSI (Hasking & Rose, 2016; Hasking, Boyes & Greves, 2018). Internal consistency was excellent in the current sample (*α* = .91).

#### 1.2.5 NSSI characteristics

NSSI characteristics were assessed with the Inventory of Statements about Self‐Injury (ISAS; Klonsky & Glenn, 2009). Lifetime history of NSSI was assessed using Section I of the ISAS. Participants were provided with a definition of NSSI, followed by questions regarding lifetime history, frequency, recent (12 months) engagement, and main method of NSSI. This section has demonstrated good test‐retest reliability (*r* = .85) and good construct validity (Kress & Hoffman, 2008). Functions of NSSI were assessed using Section II of the ISAS. Functions are divided into two higher order factors; intrapersonal (e.g., affect regulation, self‐punishment) and interpersonal (e.g., marking distress, peer bonding). Section II has demonstrated acceptable test‐retest reliability, ranging from *r* = .35 to *r* = .89 (Glenn & Klonsky, 2011). Internal consistency for each factor has also been demonstrated previously (interpersonal, *α* = .88; intrapersonal, *α* = .80; Klonsky & Glenn, 2009). Both subscales demonstrated good internal consistency among the current sample (interpersonal, *α* = .90; intrapersonal, *α* = .86). Lower order subscales for intrapersonal functions demonstrated acceptable internal consistency (intrapersonal: Affect, *α* = .73; self‐punishment, *α* = .81; anti‐dissociation, *α* = .82; anti‐suicide, *α* = .88; distress, *α* = .77). Lower order subscales for interpersonal functions also demonstrated acceptable internal consistency (interpersonal: interpersonal boundaries, *α* = .79; self‐care, *α* = .68; sensation seeking, *α* = .67; peer bonding, *α* = .85; interpersonal influence, *α* = .72; revenge, *α* = .84; autonomy, *α* = .83).

### 1.3 Procedure

Ethical approval was gained from CurtinUniversity before data were collected. Participants were sampled from the university's online research participant pool and from other universities around Australia. Participants were recruited to take part in three studies (*n* = 196; 119; 57) exploring intrapersonal and interpersonal factors associated with NSSI, and data were merged for the current analyses. Participants recruited from within the university's research participant pool were given points toward course credit. Participants recruited from other universities entered a draw to win an iPad, or one of 10 $25 gift cards in exchange for participation. Participants completed an online questionnaire, which was hosted on Qualtrics. The overarching questionnaires took approximately 45–60 min to complete. On completion, participants were given information detailing contacts for counselling assistance and information about NSSI.

### 1.4 Data analysis

Data were analysed using IBM SPSS Statistics Version 27. Although not missing completely at random [*χ* ^2^(7903) = 8236.96, *p* = .004], given the minimal missing data (<2%) across scales, expectation maximisation was used to impute missing data. A series of one‐way MANCOVAs, with appropriate follow‐up analyses, were conducted to test differences between those who had stopped self‐injuring (no self‐injury in the past 12 months) and those who had not, as well as those who wanted to stop self‐injuring and those who did not (Table 1). Interactions were tested between cessation of NSSI, and desire to stop, across all variables (Table 2). Due to the correlation between sex and a number of variables included in the analyses (Tables S1–S4), this variable was included as a covariate. Participants who identify as a sex other than male or female (*n* = 11) were excluded from the analysis due to insufficient numbers when analysing sex differences. Statistical significance was set at α ≤ .05 for main effects and interactions.

**Table 1:** Descriptive statistics and group differences on each of the variables of interest

	Have you stopped?						Do you want to stop?					
	Have not stopped	Have stopped					Do not want to stop	Do want to stop				
	*M* (CI)	*M* (CI)	*Λ*	*F*	*p*	η_p_^2^	*M* (CI)	*M* (CI)	*λ*	*F*	*P*	η_p_^2^
**DASS**			0.93	8.27	**<.001**	0.07			0.99	1.47	.222	0.01
Depression	17.85 (16.87, 18.83)	14.25 (13.11, 15.40)		22.04	**<.001**	0.06	16.41 (15.06, 17.77)	15.69 (15.03, 16.35)		0.89	.348	0.003
Anxiety	15.76 (14.90, 16.62)	13.51 (12.50, 14.52)		11.16	**.001**	0.03	14.41 (13.21, 15.60)	14.87 (14.29, 15.45)		0.47	.495	0.001
Stress	18.09 (17.25, 18.92)	15.23 (14.25, 16.21)		19.13	**<.001**	0.05	16.48 (15.32, 17.64)	16.83 (16.27, 17.40)		0.28	.595	0.001
**DERS**			0.93	3.57	**.002**	0.07			0.95	2.16	**.047**	0.05
DERS‐aware	16.22 (15.35, 17.10)	14.75 (13.50, 15.99)		3.65	.057	0.01	14.53 (13.15, 15.91)	16.44 (15.78, 17.10)		6.04	**.015**	0.02
DERS‐clarity	13.73 (12.95, 14.52)	13.56 (12.45, 14.68)		0.06	.805	<0.001	13.34 (12.10, 14.57)	13.96 (13.37, 14.55)		0.81	.368	0.003
DERS‐goals	18.64 (17.83, 19.45)	17.58 (16.42, 18.74)		2.19	.140	0.01	18.06 (16.78, 19.34)	18.16 (17.55, 18.77)		0.02	.891	<0.001
DERS‐impulse	16.91 (15.92, 17.91)	14.37 (12.95, 15.79)		8.35	**.004**	0.03	15.11 (13.54, 16.68)	16.18 (15.43, 16.93)		1.46	.228	0.01
DERS‐nonacceptance	19.05 (17.94, 20.15)	16.46 (14.89, 18.04)		7.00	**.009**	0.03	17.14 (15.40, 18.88)	18.37 (17.54, 19.20)		1.57	.211	0.01
DERS‐strategies	26.80 (25.51, 28.10)	22.75 (20.91, 24.59)		12.64	**<.001**	0.04	24.90 (22.86, 26.93)	24.66 (23.69, 25.63)		0.04	.837	<0.001
**NEQ & SEAS**			0.76	17.57	**<.001**	0.24			0.99	0.33	.920	0.01
Affect reg expectancies	12.73 (12.13, 13.33)	13.05 (12.32, 13.77)		0.45	.504	0.001	12.89 (12.05, 13.74)	12.88 (12.47, 13.29)		0.001	.976	<0.001
Negative social expectancies	12.76 (12.00, 13.51)	12.30 (11.39, 13.22)		0.57	.453	0.002	12.66 (11.59, 13.73)	12.40 (11.89, 12.91)		0.02	.663	0.001
Communication expectancies	11.34 (10.50, 12.19)	12.66 (11.64, 13.68)		3.807	.052	0.011	11.62 (10.43, 12.82)	12.38 (11.80, 12.95)		1.25	.264	0.002
Pain expectancies	13.43 (12.73, 14.13)	11.95 (11.11, 12.79)		7.01	**.008**	0.02	12.95 (11.97, 13.94)	12.43 (11.95, 12.90)		0.89	.345	0.003
Neg self‐belief expectancies	12.62 (11.93, 13.32)	12.23 (11.40, 13.07)		0.49	.483	0.001	12.65 (11.66, 13.63)	12.21 (11.74, 12.68)		0.62	.433	0.002
Self‐efficacy to resist NSSI	18.10 (16.85, 19.35)	27.86 (26.36, 29.37)		96.27	**<.001**	0.23	23.20 (21.44, 24.97)	22.76 (21.91, 23.61)		0.20	.655	0.001
**Intrapersonal total**			0.95	3.77	**.003**	0.05			0.96	2.66	**.023**	0.04
Affect regulation	7.61 (7.33, 7.88)	6.95 (6.63, 7.27)		9.21	**.003**	0.03	7.46 (7.08, 7.84)	7.10 (6.91, 7.29)		2.71	.100	0.01
Self‐punishment	7.00 (6.66, 7.33)	6.17 (5.79, 6.56)		10.13	**.002**	0.03	6.38 (5.92, 6.83)	6.79 (6.56, 7.02)		2.61	.107	0.01
Anti‐dissociation	5.34 (5.00, 5.68)	5.22 (4.83, 5.62)		0.18	.674	0.001	5.29 (4.82, 5.76)	5.27 (5.04, 5.51)		0.003	.957	<0.001
Anti‐suicide	5.09 (4.75, 5.44)	4.42 (4.03, 4.82)		6.21	**.013**	0.02	4.51 (4.04, 4.99)	5.00 (4.76, 5.24)		3.27	.072	0.01
Marking distress	5.46 (5.14, 5.79)	4.72 (4.34, 5.10)		8.57	**.004**	0.02	4.90 (4.46, 5.35)	5.27 (5.05, 5.50)		2.13	.145	0.01
**Interpersonal total**			0.97	1.35	.220	0.03			0.96	1.86	.066	0.04
Interpersonal boundaries	3.99 (3.73, 4.26)	4.01 (3.71, 4.30)		0.01	.945	<0.001	4.08 (3.73, 4.44)	3.92 (3.74, 4.10)		0.64	.426	0.002
Self‐care	4.23 (3.98, 4.49)	4.14 (3.85, 4.44)		0.22	.640	0.001	4.25 (3.90, 4.60)	4.13 (3.95, 4.31)		0.34	.560	0.001
Sensation seeking	3.84 (3.64, 4.04)	3.85 (3.62, 4.08)		0.01	.946	<0.001	4.05 (3.78, 4.33)	3.64 (3.50, 3.78)		6.84	**.009**	0.02
Peer bonding	3.19 (3.06, 3.32)	3.25 (3.10, 3.40)		0.31	.583	0.001	3.25 (3.07, 3.43)	3.19 (3.11, 3.28)		0.30	.580	0.001
Interp influence	3.94 (3.70, 4.18)	3.78 (3.51, 4.06)		0.69	.407	0.002	3.72 (3.40, 4.04)	4.00 (3.84, 4.16)		2.31	.129	0.01
Toughness	4.25 (3.99, 4.51)	4.42 (4.13, 4.72)		0.70	.403	0.002	4.46 (4.11, 4.81)	4.22 (4.04, 4.39)		1.48	.225	0.004
Revenge	3.57 (3.37, 3.78)	3.33 (3.10, 3.57)		2.31	.130	0.01	3.44 (3.16, 3.72)	3.47 (3.33, 3.61)		0.04	.851	<0001
Autonomy	3.75 (3.52, 3.98)	3.47 (3.21, 3.73)		2.60	.108	0.01	3.61 (3.30, 3.92)	3.61 (3.46, 3.77)		<0.001	.985	<0.001

*Note*: Significant *p* values are bolded. *M* = Estimated marginal means adjusting for sex and age. Sex coded as male = 1; female = 2.

**Table 2:** Have stopped NSSI × Want to stop NSSI interactions on each of the variables of interest

	*Λ*	*F*	*p*	η_p_^2^
DASS	0.98	1.79	.15	0.02
DERS	0.97	1.27	.27	0.03
NEQ & SEAS	0.99	.48	.82	0.01
Intrapersonal	0.97	1.92	.09	0.03
Interpersonal	0.95	1.96	.05	0.05

*Note*: *p < .05. Sex coded as male = 1; female = 2.

Abbreviations: DASS, Depression Anxiety and Stress Scale; DERS, Difficulties in Emotion Regulation Scale; NSSI, nonsuicidal self‐injury.

## 2 RESULTS

Two hundred and ten (56.1%) participants reported engaging in NSSI in the previous 12 months, with 164 (43.9%) reporting no NSSI in the last 12 months. Of those who had self‐injured in the last 12 months, 84 (40%) had self‐injured more than 5 times that year. Of the sample, 299 (79.9%) individuals reported a desire to stop self‐injuring, while 75 (20.1%) reported that they did not want to stop self‐injuring. Of those who wanted to stop injuring, 165 (55.2%) had engaging in NSSI in the past 12 months, and 134 (44.8%) had not engaged for at least 12 months. Of those who did not want to stop engaging in NSSI, 45 (60%) had self‐injured in the past 12 months, while 30 (40%) had not engaged for at least 12 months.

Multivariate analyses revealed several main effects differentiating individuals who had a recent history of NSSI and those who did not (Table 1). Appropriate univariate follow‐up analyses indicated that individuals who had stopped self‐injuring experienced less psychological distress (depression, anxiety, stress) than individuals who had not stopped self‐injuring; Individuals who had stopped self‐injuring had less difficulty regulating their emotions (nonacceptance, impulsivity, strategies) than individuals who had not stopped self‐injuring; NSSI was expected to be more physically painful for individuals who had not stopped self‐injuring, than those who had stopped. Individuals who had stopped NSSI had more self‐efficacy to resist engaging in the behaviour than those who had not stopped. Individuals who had stopped NSSI self‐injured less for intrapersonal reasons (affect regulation, self‐punishment, anti‐suicide, marking distress) than those who had not stopped. Main effects were also found between those who wanted to stop engaging in NSSI and those who did not want to stop, whereby individuals who did not want to stop engaging in NSSI used the behaviour more for interpersonal functions, specifically, as a means of sensation seeking. Individuals who did not want to stop engaging in NSSI also experienced less difficulties with emotion regulation, particularly awareness of emotions. No significant interactions were found (Table 2).

## 3 DISCUSSION

Research investigating the desire to self‐injure as a separate construct from having stopped self‐injuring is limited. The purpose of this study was to explore potential emotional and cognitive factors that differentiate individuals who have stopped engaging in NSSI from individuals who have not stopped, and individuals who have a desire to stop engaging in NSSI, from those who do not have a desire to stop. Identifying the differentiating factors between these groups will allow for a comparison of factors contributing to action vs desire regarding self‐injurious behaviours. Additionally, exploring differentiating factors between individuals who want to stop engaging in NSSI and individuals who do not want to stop is an important first step in identifying treatment targets for this cohort, and potentially removing barriers to the wellbeing of those who are experiencing this paradox.

Three key findings emerged from this study. First, one fifth of individuals with a history of NSSI do not wish to stop self‐injuring. As mentioned, there are several negative outcomes associated with NSSI (Andrews et al., 2013; Kiekens et al., 2018; Lewis & Mehrabkhani, 2016; Owens et al., 2016; Staniland et al., 2020). Because of the negative outcomes associated with NSSI, it is tempting to assume that individuals who engage in the behaviour want to stop. NSSI is already a highly stigmatised behaviour, and experiences of stigma are a significant barrier to help‐seeking (Rowe et al., 2014; Staniland et al., 2020). Compared to individuals who do want to stop, the fear of disclosure and help seeking may be even higher for individuals who do not wish to give up such a highly stigmatised behaviour.

Further, wanting to stop engaging in a behaviour is not the same as wanting to avoid experiencing the outcomes of the behaviour. Individuals with persistent and ongoing NSSI often increase the frequency and severity of their behaviour over time (Andrews et al., 2013). As the outcomes of NSSI get worse, a proportion of individuals may wish to avoid these outcomes, while simultaneously wanting to continue to engage in NSSI. A person‐centred recovery framework proposed by Lewis and Hasking (2021) may be beneficial for these individuals. Normalising re‐engagement and ongoing urges, identification of alternative activities/behaviours, navigating disclosures, and addressing scarring may be focal points for individuals who have little interest focusing on cessation of the behaviour itself.

A second key finding of this study suggests that holding a desire to stop engaging in NSSI does not necessarily lead to behavioural cessation. Of individuals who do want to stop, 55% had self‐injured in the past 12 months. Previous research suggests that the target goal in treatment and intervention is often cessation of NSSI (Grunberg & Lewis, 2015; Kamen, 2009; Tatnell et al., 2014). Many conceptualisations of recovery focus on abstinence of the behaviour for 6–12 months (Andrews et al., 2013; Grunberg & Lewis, 2015; Kress & Hoffman, 2008; Kruzan & Whitlock., 2019; Tatnell et al., 2014). Yet individuals with lived experience of NSSI view recovery with more nuance than cessation only; healthy relationships, emotional wellbeing, daily functioning, and self‐acceptance are just some of the many facets of recovery regarded by those with lived experience (Buser et al., 2014; Tofthagen et al., 2017; Lewis et al., 2019; Lewis & Hasking 2020; 2021). Results from the current study coincide with this more complex conceptualisation of recovery; of those who indicated that they did not want to stop the behaviour, 40% had in fact stopped for the amount of time required to categorise them as recovered by current research and clinical standards.

Lastly, researchers have repeatedly identified the main factors differentiating individuals who have stopped self‐injuring, and individuals who have not stopped (Andrews et al., 2013; Kiekens et al., 2017; Tatnell et al., 2014. Results from this study reveal that the factors differentiating individuals who have stopped from those who have not, are different from the factors differentiating individuals who want to stop from those who do not want to stop. Specifically, consistent with traditional cognitive emotional models of NSSI, individuals who are still engaging in NSSI report greater use of the behaviour for intrapersonal reasons, such as to regulate negative affect. They also report greater psychological distress, more difficulties regulating their own emotions, and lower belief in their ability to resist self‐injuring across several contexts.

In contrast, not only were the factors driving desire to stop and actioned behaviour different, but very few of the assessed factors were found to differentiate individuals who did and did not want to stop. This may reflect some of this underlying ambivalence for those who did not want to stop but had. These individuals are not self‐injuring, but view themselves in a similar way to those who are still self‐injuring. To classify 12‐month cessation as recovery neglects a significant population of people who perceive themselves as still engaging in the behaviour. Self‐injurious thoughts, psychological distress, and expectations of engaging in the behaviour appear to continue for many individuals, despite their 12‐month cessation. It is possible that NSSI was effective in the past but has recently stopped providing the same emotion regulatory capabilities it once did, and as such has not been used. Perhaps these individuals have found alternative coping strategies, while still holding onto the availability of NSSI should it be needed. Alternatively, perhaps they have not found alternative coping strategies, and are struggling to resist engaging in the behaviour. Assuming that these individuals have either “moved on” to alternative strategies, or are resisting engagement in NSSI, a critical opportunity for intervention may reside here. In this sample, not wanting to stop NSSI was associated with sensation seeking functions; interventions aimed at providing valued alternative, sensation inducing behaviours may be beneficial.

Effect sizes for factors differentiating participants having stopped self‐injuring from continuing to self‐injure ranged between small (marking distress) to large (self‐efficacy to resist NSSI). Effect sizes for factors that differentiated wanting to stop engaging in NSSI from not wanting to stop ranged between small (sensation seeking) and medium (difficulties with emotion regulation). These effects may be important in contributing to the desire to change self‐injurious behaviours, precipitating actual cessation. Taken together, these findings present an assortment of factors for clinicians to consider when working with individuals who self‐injure.

Together, these findings reveal the complexities of NSSI cessation. Inconsistencies between desire and actioned behaviour suggest that they should be treated as separate constructs, potentially driven by different factors, and should be conceptualised independently during treatment. An individual may hold a desire to change a behaviour, possibly leading to a conscious intention to do so. Yet studies exploring cessation of various behaviours including smoking (McWilliams et al., 2019) and physical activity (Rhodes & de Bruijn, 2013) indicate that intention only partially predicts behaviour change; only 30%–40% of behaviour change is explained by intention to change (Armitige & Connor, 2001; Rhodes & de Brujin, 2013). Several alternative factors may moderate this relationship, including attitude, personality, self‐efficacy, intrinsic vs extrinsic motivation, and missed opportunities, or forgetting to act on the intention (Faries, 2016; Sheeran & Webb, 2016). Such factors may be useful in explaining a proportion of the clear discrepancy between desire to stop self‐injuring, and actual behavioural cessation.

### 3.1 Theoretical implications

Many of the existing theories of NSSI recognise that there are perceived benefits for those who engage in the behaviour (Hasking et al., 2017; Hooley & Franklin, 2017; Nock 2010). The benefits and barriers model by Hooley and Franklin (2017) illustrates how a mixture of components may incentivise or deter one from self‐injuring. The model includes both internal processes (affect, self‐worth) and external influences (lack of self‐injury exposure, aversion toself‐injury stimuli such as blood or knives) to predict self‐injurious behaviour. Perhaps this expansion from purely cognitive‐emotional factors may better explain desire for/avoidance of NSSI. While it is valuable to know that benefits and barriers to NSSI can exist simultaneously, when considering behaviour, such models only allow for one of two possible outcomes (you cannot both self‐injure and not self‐injure in any given moment). However, in terms of desire it is possible to hold competing beliefs regarding both wanting and not wanting to cease the behaviour.

Ambivalence in NSSI is felt during the existence of simultaneous contradictory or opposing beliefs, feelings, or desires toward the behaviour (Gray et al., 2021; Grunberg & Lewis, 2015; Kelada et al., 2017; Norcross et al., 2011; Shaw, 2006). The inconsistencies between desire to stop and actioned behaviour in these results suggest a level of conflict, pointing toward ambivalence around individuals' experience of NSSI. Reports such as these are common among individuals who engage in NSSI; some describe confusion, frustration, and uncertainty about why they continue to engage in the behaviour when they do not want to (Tan et al., 2019), others understand their own ambivalence, describing specific reasons why they want to stop (e.g., social judgment), with an awareness that NSSI works to regulate their emotion and thy will likely engage again, despite the desire not to (Kelada et al., 2017; Shaw, 2006).

While traditional cognitive emotional models used to illustrate cessation of NSSI do not predict desire to cease the behaviour, the model of ambivalence taken from the substance use literature (Breiner et al., 1999) may be more suitable. The Ambivalence Model includes historical factors (reactivity, personality, socio‐cultural environment, personal experiences, past reinforcement); immediate factors (immediate positive and negative incentives, valued alternative behaviours); outcome expectancies, and illustrates how these factors interplay, leading to a desire for engagement in a particular behaviour, and a desire to avoid engagement in the same behaviour, simultaneously (Breiner et al., 1999). Further research is necessary to determine whether the factors in the Ambivalence Model account for the differentiation between wanting to stop, or not wanting to stop engaging in NSSI.

### 3.2 Clinical implications

Compared to individuals who wanted to stop self‐inuring, individuals who did not want to stop engaging in NSSI were more likely to report that the behaviour was a form of sensation seeking. However, many of these individuals had not self‐injured in 12 months or more. The period in which one resists engaging in NSSI, while continuing to desire its effects, may be a crucial turning point in terms of intervention. The use of alternative activities has been reported as a component of recovery for those who no longer engage in NSSI (Tofthagen et al., 2017). In a study by Tofthagen and colleagues (2017) participants deeming themselves as recovered from NSSI attributed partial success to their engagement in activities such as education, music, physical activity, breathing exercises, watching television, writing, and the formation of stable, gratifying relationships. These activities may induce sensation or “add something”, though alcohol use, drug use, and fire‐setting are also associated with both NSSI, and sensation seeking (Bresin & Mekawi, 2020; Hasking, 2017; Hasking & Claes, 2020; MacKay et al., 2009; Moran et al., 2014; Tanner et al., 2015). Individuals who do not want to stop engaging in NSSI require further consideration, both in research and in clinical practice. Interventions focused solely on emotion regulation skills may not be suitable for certain individuals, with the potential for negative consequences if their desire for sensation is overlooked.

Clinicians would benefit from understanding the factors which may motivate an individual to want to stop engaging in NSSI. It would be beneficial to further consider the emotional, cognitive, environmental, and behavioural factors associated with desire toward NSSI. Including these factors allows intervention efforts to acknowledge competing desires towards both engagement, and cessation of self‐injurious behaviours. Motivational approaches to treatment embrace ambivalence as a natural, and necessary component of decision making and behavioural change (Kress & Hoffman, 2008). Recent conceptualisations of NSSI recovery delineate conflicting desires, continued urges, and fluctuating behaviours as expected components in the multidimensional process of recovery (Lewis & Hasking, 2020). With this perspective, individuals with lived experience, personal support networks, and clinicians may understand how desire for behaviour change plays a role in treatment efforts, and create treatment goals accordingly.

### 3.3 Limitations and suggestions for future research

In the current study, the only items regarding NSSI cessation were “Do/did you want to stop self‐injury” with a binary yes/no response. Asking participants only if they want to stop may be over simplistic. Given that the current literature on NSSI recognises the existence of ambivalence (Gray et al., 2021; Kelada et al., 2017), and competing desires to stop/continue, more detailed items on this construct would be beneficial in future studies. We recommend exploring the extent to which people want to stop, and the extent to which they do not want to stop engaging in NSSI, avoiding all or nothing extremes of (a) not having no desire to stop whatsoever, or (b) having no desire to continue whatsoever.

The data used for this study were cross sectional in nature. Self‐injurious behaviours and their surrounding factors, including desire to stop, fluctuate over time (Grunberg & Lewis, 2015; Lewis et al., 2019; Whitlock et al., 2015). The factors contributing to the desire for NSSI may be best explored longitudinally. Alternatively, ecological momentary assessment (EMA) studies may capture the salience of factors at differing levels of desire. Unlike retrospective assessment which inherently includes an element of memory bias, EMA research may be used practically, providing real‐time feedback to participating individuals. EMA applications may provide information and prompts for its users, should they desire support while experiencing uncomfortable emotions (Rodriguez‐Blanco et al., 2018). This allows for data collection, while assisting individuals through short‐term fluctuations, and rapidly changing, or conflicting desires.

Additionally, the dataset used for this study included variables that were informed by previous research on the differences between individuals who had, and individuals who had not, stopped engaging in NSSI. It would be beneficial to include variables informed by research regarding desire to stop engaging in NSSI. Drawing on models of ambivalence and incorporating measures of these constructs would be important going forward. Further research could explore potential factors contributing to experiences of ambivalence, as highlighted by the current findings.

## 4 CONCLUSION

Our findings demonstrate the complexities behind NSSI recovery. Results of the current study demonstrate that active cessation of NSSI, and desire for cessation of NSSI are in fact driven by different factors. An exploration of the factors leading to differing levels of ambivalence toward engagement in NSSI will better predict whether an individual is likely to want to stop, or not want to stop their self‐injurious behaviour. Motivational approaches toward self‐injurious behaviours may highlight potential treatment targets, and resolve ambivalence toward behavioural change.

## CONFLICTS OF INTEREST

The authors declare no conflicts of interest.

## AUTHOR CONTRIBUTIONS

**Nicole Gray**: conceived and wrote the paper. **Penelope Hasking and Mark Boyes**: reviewed and contributed to writing of the paper.

## ETHICS STATEMENT

This study was approved by the Curtin University Human Research Ethics Committee. The research was conducted in accordance with the National Statement on the Conduct of Human Research and the Declaration of Helsinki. All participants provided consent to participate.

### PEER REVIEW

The peer review history for this article is available at https://publons.com/publon/10.1002/jclp.23336

## Supporting information

Supporting information.

## Data Availability

The data that support the findings of this study are available on request from the corresponding author. The data are not publicly available due to privacy or ethical restrictions.
