# Supplementary material for: Cognitive and emotional factors associated with the desire to cease non‐suicidal self‐injury
Source: J Clin Psychol. 2022 Mar 5;78(9):1896–911. doi: 10.1002/jclp.23336 (PMC9544119; doi:10.1002/jclp.23336)
Supplement: Supplementary file 1 — Supporting information. [file JCLP-78-1896-s001.docx]

| *Supplementary Table 1. Correlations Between Demographics, Variables of Interest, and DASS* | | | | | | |
| --- | --- | --- | --- | --- | --- | --- |
|  | **2.** | **3.** | **4.** | **5.** | **6.** | **7.** |
| **1. Age** | -.03 | .05 | .02 | .02 | -.04 | .05 |
| **2. Sex** | - | -.06 | -.03 | .05 | .11* | .13* |
| **3. Desire to stop** |  | - | -.04 | .07 | -.03 | -.01 |
| **4. Stopped** |  |  | - | -.39* | -.28* | -.33* |
| **5. Depression** |  |  |  | - | .69* | .73* |
| **6. Anxiety** |  |  |  |  | - | .77* |
| **7. Stress** |  |  |  |  |  | - |

** p < .05.* Sex coded as Male = 1; Female = 2

| *Supplementary Table 2. Correlations Between Demographics, Variables of Interest, and DERS* | | | | | | | | | |
| --- | --- | --- | --- | --- | --- | --- | --- | --- | --- |
|  | **2.** | **3.** | **4.** | **5.** | **6.** | **7.** | **8.** | **9.** | **10.** |
| **1. Age** | -.03 | .05 | .02 | -.03 | -.09 | -.01 | -.01 | .02 | -.001 |
| **2. Sex** | - | -.06 | -.03 | .02 | .16* | .08 | .11* | .10 | .14* |
| **3. Desire to stop** |  | - | -.04 | -.10 | -.03 | -.01 | -.08 | -.07 | -.02 |
| **4. Stopped** |  |  | - | -.14* | -.11* | -.18* | -.25* | -.26* | -.34* |
| **5. DERS-aware** |  |  |  | - | .52* | .01 | .06 | .18* | .10 |
| **6. DERS-clarity** |  |  |  |  | - | .31* | .33* | .36* | .37* |
| **7. DERS-goals** |  |  |  |  |  | - | .61* | .48* | .66* |
| **8. DERS-impulse** |  |  |  |  |  |  | - | .44* | .71* |
| **9. DERS-nonacceptance** |  |  |  |  |  |  |  | - | .64* |
| **10. DERS-strategies** |  |  |  |  |  |  |  |  | - |

** p < .05.* Sex coded as Male = 1; Female = 2

| Supplementary Table 3. Correlations Between Demographics, Variables of Interest, Outcome Expectancies, and SEAS | | | | | | | | | |
| --- | --- | --- | --- | --- | --- | --- | --- | --- | --- |
|  | **2.** | **3.** | **4.** | **5.** | **6.** | **7.** | **8.** | **9.** | **10.** |
| **1.Age** | -.03 | .05 | .02 | -.05 | .01 | .09 | -.05 | -.03 | -.07 |
| **2. Sex** | - | -.06 | -.03 | .02 | .13* | -.10 | .06 | .08 | -.06 |
| **3. Desire to stop** |  | - | -.04 | -.01 | .03 | -.06 | .07 | .07 | -.03 |
| **4. Stopped** |  |  | - | -.01 | -.02 | .12* | -.15* | -.07 | .56* |
| **5. Affect reg expectancies** |  |  |  | - | .13* | .28* | -.19* | -.07 | .09 |
| **6. Negative social expectancies** |  |  |  |  | - | -.12* | .27* | .51* | -.10 |
| **7. Communication expectancies** |  |  |  |  |  | - | -.47* | -.32* | .11* |
| **8. Pain expectancies** |  |  |  |  |  |  | - | .33* | -.09 |
| **9. Neg self-belief expectancies** |  |  |  |  |  |  |  | - | -.12* |
| **10. Self-efficacy to resist NSSI** |  |  |  |  |  |  |  |  | - |
| ** p < .05.* Sex coded as Male = 1; Female = 2 |  |  |  |  |  |  |  |  |  |

| Supplementary Table 4. Correlations Between Demographics, Variables of Interest, and Functions | | | | | | | | | | | | | | | | |
| --- | --- | --- | --- | --- | --- | --- | --- | --- | --- | --- | --- | --- | --- | --- | --- | --- |
|  | **2.** | **3.** | **4.** | **5.** | **6.** | **7.** | **8.** | **9.** | **10.** | **11.** | **12.** | **13.** | **14.** | **15.** | **16.** | **17.** |
| **1.Age** | -.03 | .05 | .02 | .13* | -.02 | .004 | -.01 | .01 | -.07 | .004 | -.03 | -.07 | -.02 | -.08 | -.02 | -.01 |
| **2. Sex** | - | -.06 | -.03 | .15* | .10 | .11* | .13* | .15* | .07 | .10* | -.12* | -.02 | .07 | -.13* | <.001 | .06 |
| **3. Desire to stop** |  | - | -.04 | .08 | -.08 | -.01 | -.10 | -.07 | .01 | -.002 | .13* | .02 | -.09 | .06 | -.01 | .004 |
| **4. Stopped** |  |  | - | -.29* | -.26* | -.08 | -.21* | -.18* | -.01 | -.12* | -.02 | .06 | -.03 | .04 | -.04 | -.12* |
| **5. Affect regulation** |  |  |  | - | .38* | .24* | .33* | .33* | .13* | .18* | -.04 | -.06 | .04 | .003 | .03 | .09 |
| **6. Self-punishment** |  |  |  |  | - | .24* | .42* | .36* | .25* | .20* | .02 | -.03 | .10 | .14* | .07 | .15* |
| **7. Anti-dissociation** |  |  |  |  |  | - | .42* | .22* | .16* | .33* | .20* | .15* | .07 | .15* | -.01 | .21 |
| **8. Anti-suicide** |  |  |  |  |  |  | - | .32* | .27* | .31* | .14* | .15* | .19* | .14* | .06 | .25* |
| **9. Marking distress** |  |  |  |  |  |  |  | - | .43* | .39* | .19* | .10 | .55 | .31 | .35* | .33* |
| **10. Interpersonal boundaries** |  |  |  |  |  |  |  |  | - | .37* | .32* | .31* | .44* | .37* | .50* | .50* |
| **11. Self-care** |  |  |  |  |  |  |  |  |  | - | .40* | .31* | .40* | .28* | .26* | .45* |
| **12. Sensation seeking** |  |  |  |  |  |  |  |  |  |  | - | .41* | .29* | .46* | .22* | .40* |
| **13. Peer bonding** |  |  |  |  |  |  |  |  |  |  |  | - | .34* | .28* | .34* | .38* |
| **14. Interpersonal influence** |  |  |  |  |  |  |  |  |  |  |  |  | - | .25* | .56* | .29* |
| **15. Toughness** |  |  |  |  |  |  |  |  |  |  |  |  |  | - | .22* | .50* |
| **16. Revenge** |  |  |  |  |  |  |  |  |  |  |  |  |  |  | - | .33* |
| **17. Autonomy** |  |  |  |  |  |  |  |  |  |  |  |  |  |  |  | - |

** p < .05.* Sex coded as Male = 1; Female = 2
